# A Glucosamine-Specific Lectin from Green Dragon No. 8 Beans (*Phaseolus vulgaris*) Induced Apoptosis on Nasopharyngeal Carcinoma Cells

**DOI:** 10.1155/2015/760539

**Published:** 2015-07-28

**Authors:** Yau Sang Chan, Lixin Xia, Tzi Bun Ng

**Affiliations:** ^1^State Key Laboratory of Respiratory Disease for Allergy at Shenzhen University, School of Medicine, Shenzhen University, Nanhai Avenue 3688, Shenzhen, Guangdong 518060, China; ^2^Shenzhen Institutes of Advanced Technology, Chinese Academy of Sciences, School of Medicine, Shenzhen University, Nanhai Avenue 3688, Shenzhen, Guangdong 518060, China; ^3^School of Biomedical Sciences, The Chinese University of Hong Kong, Lo Kwee Seong Integrated Biomedical Sciences Building, Shatin, New Territories, Hong Kong

## Abstract

A lectin exhibiting antiproliferative activity on tumor cell lines but devoid of antifungal activity has been purified from *Phaseolus vulgaris* cv. Green Dragon no. 8 seeds. The lectin was a 60 kDa dimeric protein with two 30 kDa subunits. It was a glucosamine-specific lectin as implied from the inhibitory effect of glucosamine on hemagglutinating activity of the lectin. The steps for isolation of the lectin involved Affi-gel blue gel (affinity gel), Mono Q (anion exchanger), and Superdex 75 column (size exclusion). The lectin was purified 20.8-fold from the crude extract of the beans. The purified lectin showed antiproliferative activity on breast cancer MCF7 cell line and nasopharyngeal cancer HONE1 and CNE2 cell lines, but a low activity on normal skin fibroblast HSF98 cell line. The lectin was shown to induce apoptosis on HONE1 cells, as indicated by increased phosphatidylserine externalization and mitochondrial depolarization. It also blocked HONE1 cell division and kept the cells at the G_2_/M phase of the cell cycle.

## 1. Introduction

Legumes are commonly used in cuisines worldwide. Especially for vegetarians, legumes act as an important part of their diet as they can provide a good source of proteins [1]. Most cooked legume proteins lose their biological activity and are digested and absorbed by our body [2]. In fact, many proteins with a variety of bioactivities are present in the raw beans. For example, some of them (e.g., defensins) can inhibit the growth of pathogenic fungi [3], and some of them (e.g., trypsin inhibitors) exhibit anti-insect activity [4]. In addition to defense against pathogens and predators, some of the bean proteins (e.g., phytohemagglutinins) also exert antiproliferative activities on cancer cell lines [5]. With such a diversity of bioactivities, bean proteins have aroused the interest of numerous researchers.

Kidney bean (*Phaseolus vulgaris*) is a major member of the family of leguminous plants. It is grown worldwide and is developed into many different cultivars. A number of the beans (e.g., red kidney bean, black beans) contain lectins [6, 7], and lectins from different cultivars exhibit different types of biological activities, to different extents [8]. For example, lectin from brown kidney beans but not in that of the Indian cultivar beans exhibited antiproliferative activity on tumor cell lines [9, 10]. We may miss some lectins with potent biological activities from* P. vulgaris* cultivars that have not been investigated, and it is possible to seek new lectins with potential applications. Recently, we have detected the presence of a lectin from a new* P. vulgaris* cultivar, Green Dragon no. 8 beans, which has not been studied before. Here we tried to isolate the lectin from the beans and to study the biological activities in this newly identified lectin.

## 2. Materials and Methods

### 2.1. Isolation of Lectin from Green Dragon No. 8 Beans

The beans were soaked in distilled water overnight, homogenized in a blender, and centrifuged twice at 30,000 g at 4°C for 25 min. The supernatant was collected as crude extract of the beans. The extract was loaded onto an Affi-gel blue gel column preequilibrated with 10 mM Tris-HCl (pH 7.6) buffer. The unadsorbed materials were discarded, and the adsorbed fraction was collected by elution with 1 M NaCl in 10 mM Tris-HCl (pH 7.6) buffer. The fraction was dialyzed thoroughly in double-distilled water and lyophilized. It was resuspended in 10 mM Tris-HCl (pH 7.6) buffer and loaded onto a Mono Q column through FPLC using an AKTA purifier. The unadsorbed materials were discarded. The adsorbed materials were eluted using a 0 to 1 M NaCl gradient. The eluted fractions in the major absorbance peak containing the lectin were collected, dialyzed, lyophilized, and subjected to FPLC-gel filtration on a Superdex 75 column. The major absorbance peak eluted constituted purified lectin from the Green Dragon no. 8 beans [11].

### 2.2. Assay of Hemagglutinating Activity

Twofold serial dilution of protein sample was performed using 50 *μ*L PBS in a 96-well U-plate, followed by addition of 50 *μ*L 2% rabbit red blood cells. The plate was incubated for 1 hr to allow the cells of the control (PBS only) to sink to the bottom and appear as a red spot. Hemagglutinating activity causes cell aggregation, resulting in a plaque of cells in the well [12].

### 2.3. Sugar Specificity Test

Twofold serial dilution of protein sample was performed using 50 *μ*L PBS containing 500 mM solutions of different carbohydrates, and assay of hemagglutinating activity was performed. Competitive inhibition causes reduction of hemagglutinating activity of the protein. Then, twofold serial dilution of protein sample was performed using 50 *μ*L PBS containing different concentrations of the specific carbohydrate, and assay of hemagglutinating activity was performed to deduce the strength of inhibitory effect of the carbohydrate [13].

### 2.4. Sodium Dodecyl Sulphate Polyacrylamide Gel Electrophoresis

Protein sample was added to loading buffer containing *β*-mercaptoethanol and boiled for 10 min for denaturation. The sample was loaded onto a 15% polyacrylamide gel, and SDS-PAGE was performed at constant voltage of 120 V for 80 min. The gel was stained with Coomassie Brilliant Blue for 1 hr and destained with 10% acetic acid overnight [14].

### 2.5. MTT Assay

Breast cancer MCF7 cells were purchased from American Type Culture Collection (ATCC). A poorly differentiated Epstein-Barr virus (EBV) negative nasopharyngeal squamous carcinoma CNE2 cell line was purchased from the Sun Yat-Sen University of Medicinal Sciences, Guangzhou, China. Another poorly differentiated EBV positive nasopharyngeal squamous carcinoma cell line raised from another nasopharyngeal carcinoma (NPC) patient, HONE1, was generously provided by the Department of Anatomy, The University of Hong Kong. Human skin fibroblast HSF98 cells were provided by the Department of Obstetrics and Gynaecology, The Chinese University of Hong Kong. MCF7, HONE1, CNE2, and HSF98 cells were seeded onto a 96-well plate overnight, and different concentrations of the lectin were added and the mixture was incubated for 24, 48, or 72 hr. The media were removed, and 25 *μ*L of 5 mg/mL MTT in PBS was added to the cells and incubated for 4 hr. Then 150 *μ*L DMSO wells were added and OD 580 nm was read using a microplate reader [15].

### 2.6. Flow Cytometry

HONE1 cells were seeded onto a 6-well plate overnight, and different concentrations of the lectin were added for a 48 hr treatment. The cells were trypsinized and washed with PBS. For Annexin V-FITC and PI staining, 250 *μ*L binding buffer containing 1.25 *μ*L Annexin V-FITC and 0.5 *μ*L PI were added to the cells and incubated in the dark for 15 min. For JC-1 staining, 250 *μ*L of PBS containing 2.5 *μ*g/mL JC-1 was added to the cells and incubated in the dark for 15 min. Cells were analyzed by a BD LSRFortessa Cell Analyzer [16].

## 3. Results

**Figure 1:**
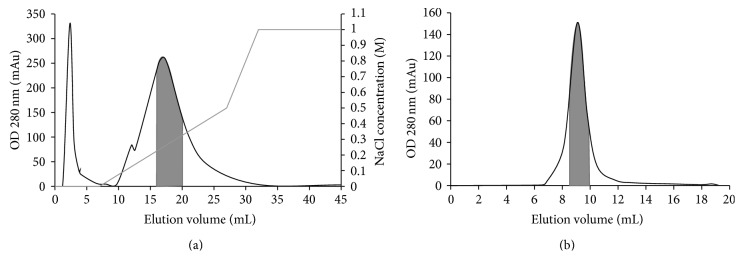
Profile of elution on purification of the lectin from Green Dragon no. 8 beans on (a) Mono Q column and (b) Superdex 75 column. The shaded area represents the fractions that constituted the lectin.

**Figure 2:**
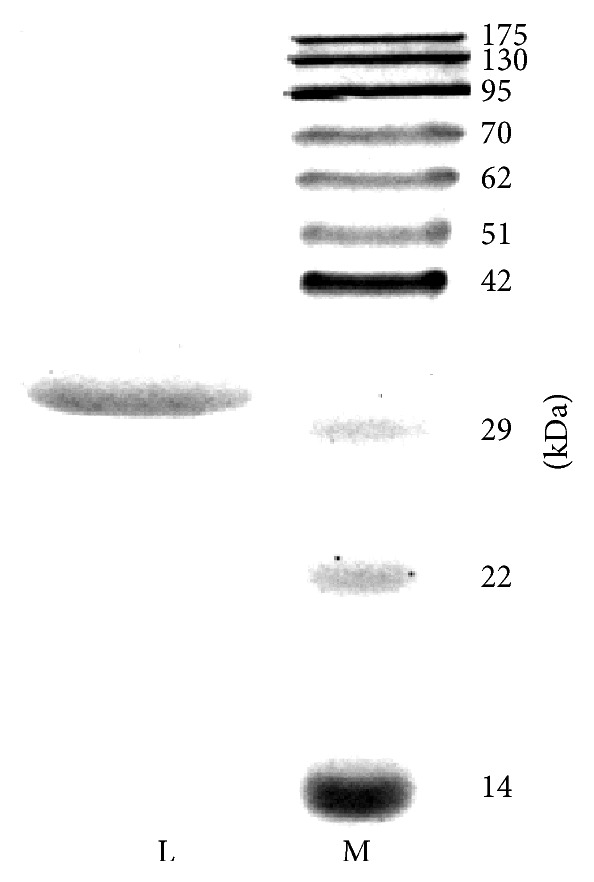
Results of SDS-PAGE on the lectin. Lane L: lectin presented in the target fraction eluted from Superdex 75. Lane M: protein ladder.

Isolation of the lectin from Green Dragon no. 8 beans involved three chromatographic steps. Crude extract of the beans underwent affinity chromatography on Affi-gel blue gel. The lectin was adsorbed on the gel, allowing removal of majority of the beans' pigments that were eluted as the flowthrough. The bound fraction with the lectin underwent FPLC-anion exchange chromatography on Mono Q. At pH 7.6, a pH value probably higher than the isoelectric point of the lectin, the lectin was negatively charged and adsorbed onto the column and was eluted at around 0.25 M NaCl (Figure 1(a)). The fraction underwent FPLC-gel filtration on Superdex 75, and the lectin was eluted at around the 9th mL (Figure 1(b)). The calibration curve of the column revealed the molecular size of the lectin to be around 60 kDa. The fraction yielded a 30 kDa band on SDS-PAGE (Figure 2). Therefore, the lectin should be a dimeric 60 kDa protein. The above purification protocol yielded around 68 mg lectin from 100 g beans and achieved a 20.8-fold purification (Table 1).

**Figure 3:**
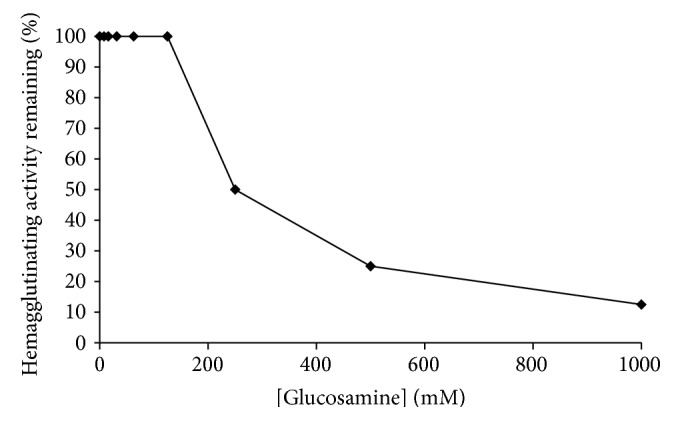
Effect of glucosamine on the hemagglutinating activity of the lectin.

Carbohydrate specificity test through competing inhibition of the hemagglutinating activity of the lectin toward red blood cells revealed that the lectin was glucosamine specific, but not specific toward glucose, mannose, galactose, rhamnose, maltose, arabinose, N-acetyl-galactosamine, *α*-methyl-pyranoside, raffinose, mannitol, and xylitol. Besides, 250 mM glucosamine was required to give detectable inhibition of the hemagglutinating activity (Figure 3).

**Figure 4:**
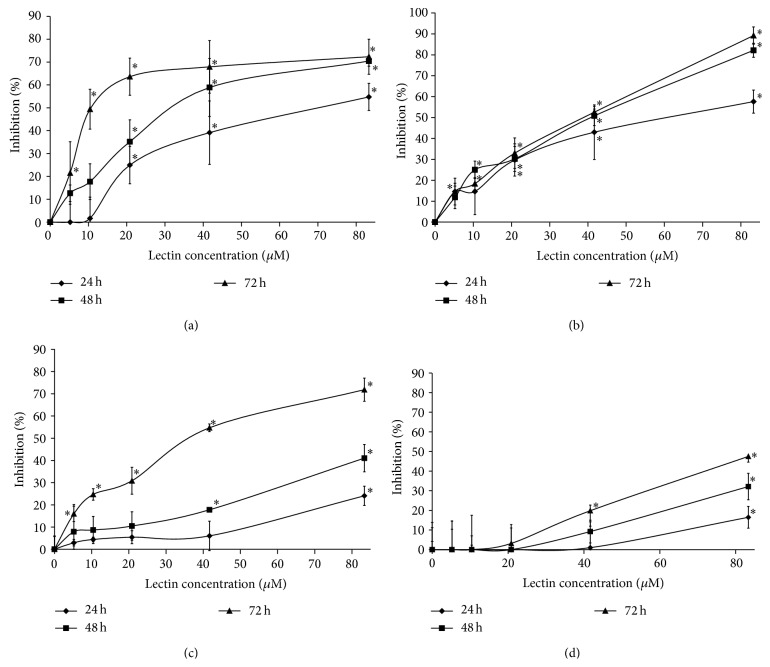
Results of MTT assay on (a) MCF7 cells, (b) HONE1 cells, (c) CNE2 cells, and (d) HSF98 cells upon treatment of the lectin for time intervals. Data represent mean ± SD (*n* = 3). Data points marked with *∗* represent *p* < 0.05.

Green Dragon no. 8 bean lectin exhibited antiproliferative activity on several tumor cell lines. It inhibited breast cancer MCF7 cells after treatment for 24, 48, and 72 hr with IC_50_ values of 65.2 *μ*M, 33.9 *μ*M, and 15.7 *μ*M, respectively (Figure 4(a)). It also inhibited nasopharyngeal carcinoma HONE1 cells after treatment for 24, 48, and 72 hr with IC_50_ values of 62.3 *μ*M, 31.6 *μ*M, and 28.7 *μ*M, respectively (Figure 4(b)). It also slightly affected nasopharyngeal carcinoma CNE2 cells, after treatment for 24 and 48 hr. 80 *μ*M of the lectin caused around 20% and 40% reduction of the cell viability, while a 72 hr treatment of the lectin raised a more obvious inhibitory effect with an IC_50_ value of 34.6 *μ*M (Figure 4(c)). On the other hand, the antiproliferative activity of the lectin on normal human skin fibroblast HSF98 cells was much lower compared with that on the aforementioned tumor cell lines. A slight inhibition could be observed on the cells until the lectin concentration was raised to 80 *μ*M under a 24 and 48 hr treatment. Also, the IC_50_ value under a 72 hr lectin treatment was also beyond 80 *μ*M (Figure 4(d)). This indicated the relative low toxicity of Green Dragon no. 8 lectin on the normal cell line, while the lectin was effective in inhibition of tumor cell lines.

**Figure 5:**
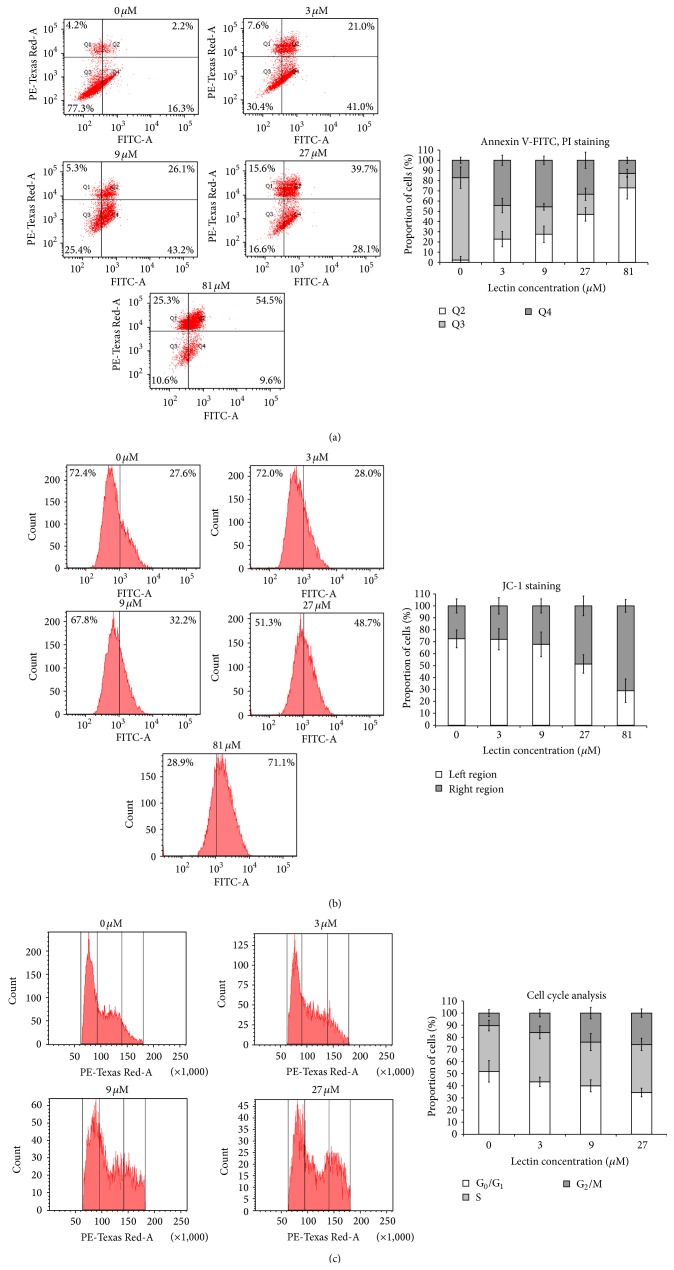
Results of flow cytometry on (a) Annexin V-FITC staining, (b) JC-1 staining, and (c) cell cycle analysis on HONE1 cells treated with the lectin for 48 hr. The bar charts represent proportion of cells located on each of the regions. Data represent mean ± SD (*n* = 3).

Green Dragon no. 8 bean lectin induced signs of apoptosis on HONE1 cells which were detected by flow cytometry. Upon Annexin V-FITC and PI staining of the cells treated with increasing concentrations of the lectin, there was shifting of the cells from the lower left quadrant (Q3) to the right (Q4), indicating increasing intensity of green fluorescence signal from Annexin V-FITC, which implied an increase in Annexin V binding to phosphatidylserine, which is exposed on the cell surface during early apoptosis. The amount of cells at the upper right quadrant (Q2) also had increased after treatment with increasing concentrations of the lectin, which indicated that more cells were entering late apoptosis or dying under the lectin's activity (Figure 5(a)). Upon JC-1 staining, the cells treated with increasing concentration of the lectin showed escalating intensity of green fluorescence signal. JC-1 is membrane permeable and positively charged and tends to accumulate in the electronegative interior of mitochondria, where it can form aggregates that emit red fluorescence. It emits red fluorescence in healthy cells with normal mitochondrial potential. Under certain conditions (e.g., apoptosis), opening of the mitochondrial permeability transition pores allows ion passage and causes disruption of mitochondrial potential. The change in ionic status caused JC-1 to stay in the monomeric form that emits green fluorescence. Upon JC-1 staining, healthy cells should present a low level of green fluorescence, while apoptotic cells probably show increased green fluorescence. Treatment of Green Dragon no. 8 bean lectin caused an increase in green fluorescence intensity, indicating that the lectin could cause mitochondrial depolarization during induction of apoptosis in HONE1 cells (Figure 5(b)). After PI staining of fixed lectin-treated HONE1 cells, the majority of cells at the first intensity peak at the left were at G_0_/G_1_ phase, while the cells at G_2_/M phase had doubled genetic materials and had doubled fluorescence intensity, and the cells at S phase had the intensity between the two peaks. After treatment with higher lectin concentration, the proportion of cells at G_0_/G_1_ phase had decreased, while that at G_2_/M phase had increased (Figure 5(c)). MTT assay implied that the cell viability had decreased upon the treatment. Thus the lectin probably did not induce HONE1 cell proliferation but blocked the cells from dividing and kept them at the G_2_/M phase.

## 4. Discussion

Green Dragon no. 8 beans were purchased from a store in Guangdong, China. Mainland China is one of the countries that produce most kidney beans in the world, and a variety of* P. vulgaris* cultivars can be found. Besides the most well-studied, common ones, for example, red kidney beans, that are grown worldwide, there are some rare cultivars, for example, Chinese pinto beans and blue tiger king beans, which are only found in China [17, 18]. Green Dragon no. 8 bean is also a new, rarer* P. vulgaris* cultivar that is only present there. We established the first study on the proteins in this bean.

The purified lectin from Green Dragon no. 8 beans was found to be a 60 kDa glucosamine-binding lectin. Though having similar molecular sizes to most of other* P. vulgaris* lectins, it has a relatively uncommon sugar specificity among the various lectins, together with Chinese pinto bean lectin and brown kidney bean lectin [9, 17]. Green Dragon no. 8 lectin had lower specific hemagglutinating units (8356 unit/mg) than that of Chinese pinto bean (202735 unit/mg) and brown kidney bean (32649 unit/mg) [9, 17]. It also required a much higher concentration of glucosamine (250 mM) than Chinese pinto bean lectin (20 mM) and brown kidney bean lectin (12.5 mM) to induce the hemagglutinating activity toward rabbit red blood cells [9, 17]. All these 3 lectins could induce antiproliferative effect on nasopharyngeal carcinoma cell lines. A 48 hr treatment of Green Dragon no. 8 lectin on HONE1 cells resulted in an IC_50_ value of 31.6 *μ*M, while Chinese pinto bean lectin gave rise to an IC_50_ value of 17.3 *μ*M [17]. On the other hand, a 48 hr treatment of 80 *μ*M Green Dragon no. 8 lectin on CNE2 cells caused only ~40% inhibition on the cells, while similar treatment of brown kidney bean lectin could achieve an IC_50_ value of 6.64 *μ*M [9]. The glucosamine-binding capability of brown kidney bean lectin was found to be crucial to its antiproliferative activity. It is highly possible for Green Dragon no. 8 lectin to show similar characteristics. Compared with the other two lectins, Green Dragon no. 8 lectin has a milder hemagglutinating activity as well as antiproliferative activity. A high hemagglutinating activity from raw, active form of the lectin could cause poisoning as the lectin can bind onto the intestinal cells [19]. Green Dragon no. 8 lectin, having a lower specific hemagglutinating activity, may show a lower side effect than the other two lectins during intake into the body.

Though Chinese pinto bean lectin and brown kidney bean lectin had potent antiproliferative activity on nasopharyngeal carcinoma cell lines, the studies of the effects of these two lectins on the cancer cells were not in depth. Here we found that Green Dragon no. 8 lectin induced apoptosis on HONE1 cells, as detected by phosphatidylserine externalization and mitochondrial depolarization, as well as cell cycle arrest at G_2_/M phase. These observations showed the effectiveness of the lectin to suppress the growth of nasopharyngeal carcinoma. There were very few reports on the use of lectins to inhibit this type of tumor. This report, together with the previous study on the Chinese pinto bean lectin and brown kidney bean lectin [9, 17], may give an insight into the application of glucosamine-specific lectins to treat nasopharyngeal carcinoma.

The various parts of the plant* Phaseolus vulgaris* L. commonly known as the kidney bean have been employed in Ayurvedic and Unani practice in India for antidiabetic therapy [20, 21].* Phaseolus vulgaris *can also be used for 11 inducing weight loss [22, 23]. We have shown herein that it has anticancer effects.

## 5. Conclusion

Green Dragon no. 8 beans contained a glucosamine-binding lectin that could induce apoptosis on nasopharyngeal carcinoma cell lines. Similarly, some other glucosamine-binding lectins (Chinese pinto bean lectin and brown kidney bean lectin) also exhibited antiproliferative effects on those cells. Application of glucosamine-binding lectins may be an approach of nasopharyngeal cancer treatment.

## Figures and Tables

**Table 1:** Table of purification of the lectin from Green Dragon no. 8 beans.

	Yield (mg/70 g beans)	Specific hemagglutinating activity (unit/mg)	Total hemagglutinating activity (10^5^ units)	% recovery	Fold of purification
Crude extract	8800	402	35.39	100	1
Affi-gel blue gel	828	2731	22.61	63.9	6.8
Mono Q	147.7	4915	7.26	20.5	12.2
Superdex 75	47.5	8356	3.97	11.2	20.8
